# Impacts of Wild Pigs on Space Use and Movements of Wild Turkeys During Autumn and Winter

**DOI:** 10.1002/ece3.71403

**Published:** 2025-05-06

**Authors:** Travis E. Stoakley, Stephen J. Zenas, Vienna R. Brown, Mark D. Smith, William D. Gulsby, Bret A. Collier, Stephen S. Ditchkoff

**Affiliations:** ^1^ College of Forestry, Wildlife and Environment Auburn University Auburn Alabama USA; ^2^ United States Department of Agriculture, Animal and Plant Health Inspection Service, Wildlife Services, National Feral Swine Damage Management Program National Wildlife Research Center Fort Collins Colorado USA; ^3^ School of Renewable Natural Resources Louisiana State University Baton Rouge Louisiana USA

**Keywords:** interspecific interaction, movement ecology, perceived risk, spatial interaction, wild pig, wild turkey

## Abstract

Wild pigs ( *Sus scrofa* ) affect native flora and fauna in the areas they invade, including ground‐nesting birds. While results from camera‐based studies have suggested that wild pigs could spatiotemporally impact resource selection of wild turkeys ( *Meleagris gallopavo* ), there has yet to be published a foundational mechanism proposed to support such impact. Additionally, while the autumn and winter seasons serve as an important period for improving body condition for spring breeders like wild turkeys, there exists a knowledge gap in the literature with respect to potential impacts of wild pigs on wild turkeys during this non‐breeding period. We proposed a novel methodology for exploring the spatiotemporal relationship between wild pigs and wild turkeys through the co‐employment of a camera survey to estimate wild pig activity and GPS units to monitor wild turkey space use. Our study monitored 12 wild turkeys across a 9000‐ha study area in east‐central Alabama during the autumn and winter seasons. We hypothesized that wild turkeys would exhibit reduced use and altered movement rates in areas with greater wild pig activity. Our results suggested that wild turkeys displayed slower rates of movement and had lower predicted probabilities of daytime use and roost site selection in areas of greater wild pig activity. Our study was limited to one season, but paired with previous camera‐based studies, we propose that wild pigs could serve as a perceived disturbance risk to wild turkeys, leading to avoidance of areas with greater wild pig activity. We also believe wild pigs could compete with wild turkeys for hard mast, which could explain the negative relationship between wild pig activity and predicted probabilities of daytime use among female wild turkeys in hardwood and riparian areas. Our study showcases the potential value in pairing multiple spatiotemporal data types (e.g., GPS‐data and camera‐based estimates) in future interspecific wildlife research.

## Graphical abstract

We tested a novel methodology to evaluate the relationship between wild pig activity and wild turkey space use in Alabama during the autumn and winter seasons. We found that wild turkeys displayed an avoidance of wild pigs during the daytime and when selecting roost sites and moved at slower rates in areas of greater wild pig activity, which we attributed to a perceived disturbance risk and competition for hard mast. Future interspecific wildlife research could benefit from the cooperation of multiple data types to examine spatiotemporal relationships.

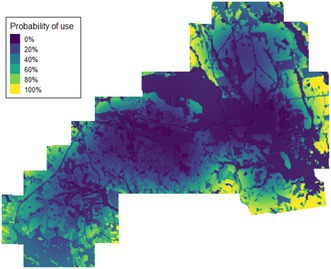

## 1 Introduction

Wild pigs ( *Sus scrofa* ) are an invasive introduced species in North America with a wide range of documented environmental and ecological impacts (Ditchkoff and Bodenchuk 2020; McDonough et al. 2022). Over the past three decades, populations of wild pigs have saturated the southeastern U.S. and are currently present in every county in Alabama, Arkansas, Florida, Georgia, Mississippi, and South Carolina (USDA 2024). Wild pigs negatively impact native species through direct predation (Jolley et al. 2010), competition for resources (Fay et al. 2023), and destruction of habitat (Strickland et al. 2020). There is also evidence that wild pigs may impact native species through perceived disturbance risk and resource partitioning (Hegel et al. 2019; Osugi et al. 2019). Responses to perceived risk can be measured by the magnitude of effect that potential encounters have on movement patterns (Sabal et al. 2020). Resource partitioning explains the mechanism by which the competition between species for resources such as space and forage leads to a division of use (Walter 1991).

There has been particular concern for wild pigs regarding impacts on ground‐nesting birds (McDonough et al. 2022). Some artificial nest studies have speculated that wild pigs may impact the nesting success of gallinaceous birds such as northern bobwhite ( *Colinus virginianus* ) and wild turkeys ( *Meleagris gallopavo* ; Tolleson et al. 1993; Sanders et al. 2020; McInnis 2021). Additionally, Carpio et al. (2023) examined fecal samples of wild pigs and found evidence of direct consumption of red‐legged partridge ( *Alectoris rufa* ), a gallinaceous bird of conservation concern in Spain. Several camera‐based studies have found a negative relationship between activity indices of wild pigs and temporal use patterns by wild turkeys (Lewis et al. 2022; Walters and Osbourn 2022; Smith 2023; McDonough et al. 2024). While the results of these studies have suggested a potential spatial aspect of this relationship, there is currently a lack of applied methodologies to quantify the impacts of wild pigs on resource selection of wild turkeys or an understanding of the mechanisms that drive such impacts.

The role of seasonality in how wild pigs affect wild turkeys and other native species is also poorly understood. However, some evidence suggests that wild pigs may compete with or temporally shift the resource use of native species during the autumn and winter seasons. Fay et al. (2023) conducted a camera survey baited with acorns during autumn and winter (September–February) and found that wild pigs were significant competitors for hard mast. Additionally, Dykstra et al. (2023) conducted an autumn camera survey (September–November) and found that raccoons ( *Procyon lotor* ) and eastern gray squirrels ( *Sciurus carolinensis* ) shifted temporal activity patterns when wild pigs were present. Forage availability during the autumn and winter seasons plays a critical role in the condition and reproductive success of spring breeders like wild turkeys (Wunz and Hayden 1975; Porter et al. 1980; Vander Haegen et al. 1988; McShea and Healy 2003). Therefore, interspecific interactions that limit or reduce access to food resources during autumn and winter have the potential to substantially impact productivity.

Hard mast like acorns, pulse resources that are primarily constrained to hardwood stands, are important in the nutrition of wild turkeys during autumn and winter such that resource selection has been shown to shift from hardwood areas during periods of low acorn availability (Gardner and Arner 1968; Porter et al. 1983; Rumble and Anderson 1996; Nguyen et al. 2004). Wild pigs are known competitors of wild turkeys for hard mast (McDonough et al. 2022; Fay et al. 2023), so great activity by wild pigs or years of low acorn abundance could reduce wild turkey use of hardwood areas (Wood and Barrett 1979; McDonough et al. 2024), with potential negative implications on condition, reproduction, and survival in the following spring (Vander Haegen et al. 1989; Roberts et al. 1995; Burhans et al. 2000; Lehman et al. 2010; Baici and Bowman 2023).

Our study objective was to test a novel methodology of adjoining camera survey data for wild pigs with GPS‐movement data of wild turkeys during the autumn and winter seasons (1 October 2022–1 January 2023) to quantify the potential spatiotemporal relationship between wild pig activity on movements and space use of wild turkeys. We applied a use‐availability resource selection framework to analyze activity indices of wild pigs across the study area in relation to movement metrics of wild turkeys (e.g., step length, daytime selection, and roost site selection; Johnson 1980). We hypothesized that areas with greater wild pig activity would be associated with greater step lengths and less roost site selection. We also hypothesized that we would observe less daytime selection of areas with greater wild pig activity, particularly in hardwood stands where competition for hard mast would be the greatest. Changes in rates of movement or reduced use of areas with greater wild pig activity could indicate a potential spatiotemporal avoidance behavior of wild turkeys by wild pigs.

## 2 Study Area

**FIGURE 1:**
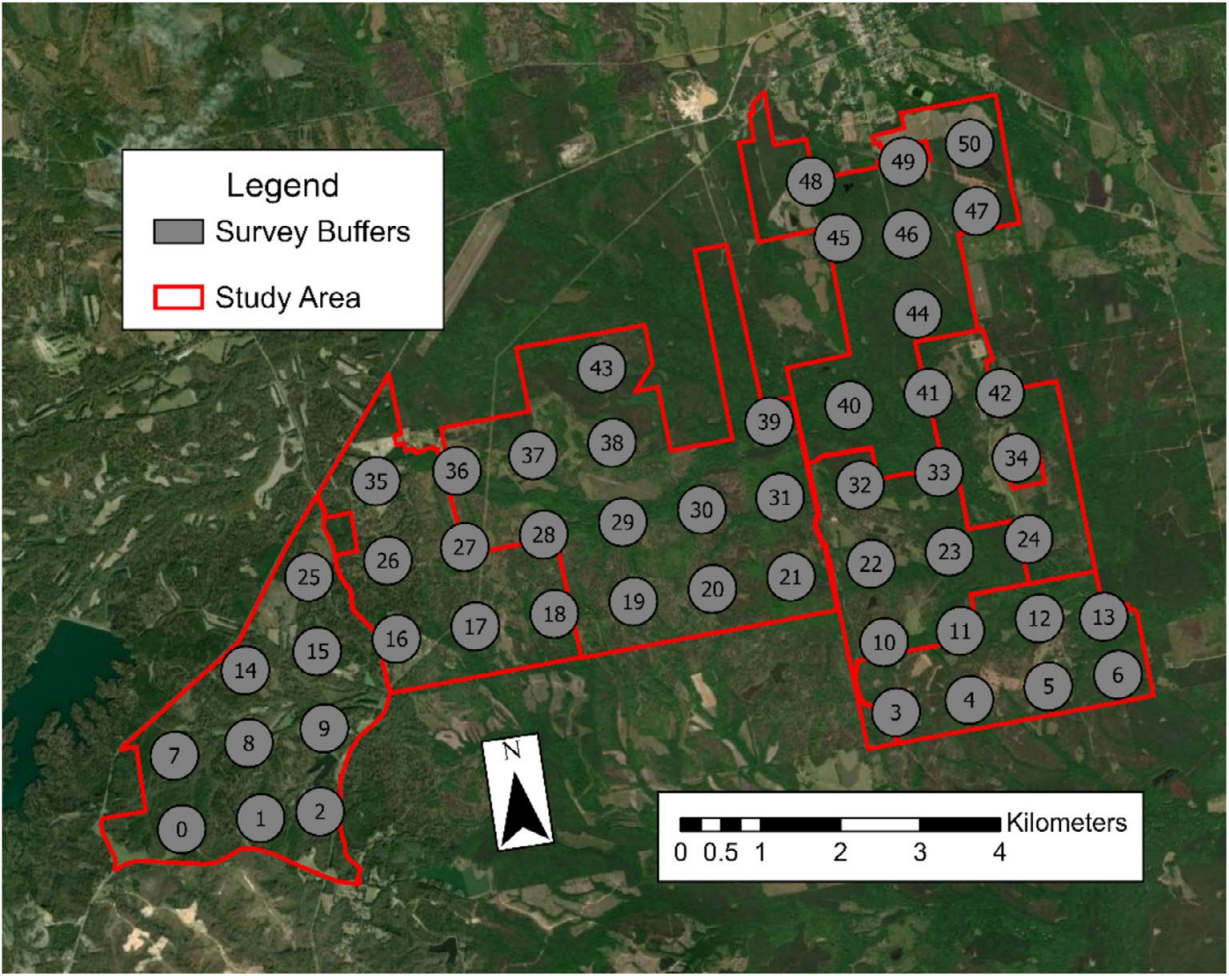
Study area map of seven adjoining private properties in east‐central Alabama with survey buffers shaded gray and study area bounds outlined in red. The southeastern property (survey buffers 3–6 and 12, 13) was surrounded by a 2.5 m fence and free from wild pigs.

Our study was comprised of seven contiguous private properties (9186 ha total) in east‐central Alabama. The region was characterized by warm, wet winters and hot, humid summers typical of the southeastern U.S. (average temperature = 18°C; annual rainfall ≈133 cm; Long 1974). The study area consisted of 1823 ha of open cover [9.9%] and 5562 ha of forest cover [60.5%] (3333 ha pine [36.3%], 1330 ha hardwood [14.5%], and 899 ha mixed pine‐hardwood [9.8%]). The properties were managed for timber production (longleaf [ *Pinus palustris* ] and loblolly pine [ *P. taeda* ]) and wildlife game species (white‐tailed deer [ *Odocoileus virginianus* ], northern bobwhite [ *Colinus virginianus* ], mourning dove [ *Zenaida macroura* ], and wild turkey). Among the pine‐dominated landscape were intermixed hardwood stands of oaks (*Quercus* spp.), hickory (*Carya* spp.), maple (*Acer* spp.), and elm (*Ulmus* spp.) (Godfrey 1988; Samuelson 2020). The 476‐ha property in the southeastern corner of the study area (camera locations 3–6 and 10–12) was enclosed by a 2.5‐m fence and free from wild pigs (Figure 1).

## 3 Methods

### 3.1 GPS‐Unit Deployment on Wild Turkeys

We deployed 12 backpack‐style GPS‐VHF units (Lotek UK Ltd., Wareham, UK) and aluminum rivet leg bands (National Band and Tag Co., Newport, KY, USA) on wild turkeys (i.e., five males and seven females) between January and March 2022. Individuals were captured with rocket nets over areas baited with cracked corn (see Bakner et al. 2023) with handling procedures approved by Auburn University IACUC (PRN: 2021‐3994). We determined the age class of individuals by the presence of barring on the ninth and tenth primary feathers, and sex was differentiated by breast feather coloration (Pelham and Dickson 1992). The GPS‐VHF units collected locations from 1 October 2022 to 1 January 2023. Daytime locations were recorded by GPS‐VHF units every 2 h between 0800 and 1600, with an additional location recorded at 0000 for the nighttime roost site. We monitored wild turkeys weekly during the 1 October 2022 to 1 January 2023 study period with Yagi antennas and GPS‐downloading devices.

### 3.2 Camera Survey for Wild Pigs

We conducted a camera survey in mid‐October 2022 to estimate activity indices of wild pigs across the study area according to Lewis et al. (2022) and McDonough et al. (2024). We applied a 1‐km^2^ grid over the study area in ArcGIS Pro (Esri, Redlands, CA, U.S.) to determine locations for camera deployment and excluded grid cells that were < 25% within the bounds of the study area. We placed a camera (Reconyx PC800 Hyperfire Professional IR Cameras; Reconyx Inc., Holmen, WI, U.S.) within a 300‐m radius buffer of the center of each of the 51 unique 1‐km^2^ grid cells (Figure 1). The camera spacing of 1‐km^2^ was used because it was less than the home range size of wild pigs that were monitored on the study site ($\bar{x}$ = 3.45 km^2^, Gomez‐Maldonado n.p.). The camera survey was divided into two phases: a 1‐week pre‐baiting phase and a 1‐week camera deployment phase. For the 1‐week pre‐baiting phase, we baited each camera site with 11 kg of whole corn 1 week prior to camera deployment, with an additional 11 kg of whole corn rebaited 3–4 days later to maintain continuity of bait availability. For the 1‐week camera deployment phase, at each camera site, we deployed cameras and baited with 11 kg of whole corn, with an additional 11 kg of whole corn rebaited 3–4 days later. Cameras were oriented north–south on trees to avoid sun exposure (e.g., shadow triggers) and were placed 1 m from the ground and 5 m from bait with visual obstructions removed. We programmed cameras to take three images upon detection of motion with a 1‐min buffer period between triggers. We removed cameras after the 1‐week deployment phase (Williams et al. 2011).

### 3.3 Activity Estimates of Wild Pigs

We manually tagged images of wild pigs in Program TimeLapse2 V2.2.3.9 (University of Calgary, Calgary, CA) for each of the 51 camera sites. Individual wild pigs were differentiated by size, sex, pelage, unique physical characteristics, sounder association, and non‐overlapping timing of visitation (Williams et al. 2011; Gomez‐Maldonado et al. 2024). Total counts at each grid were determined as the total number of unique individual wild pigs per respective camera. Home ranges of wild pig sounders in the southeastern U.S. are typically larger ($\bar{x}$ = 4.75 km^2^ for Clontz et al. 2021; 3.4 km^2^ for Kay et al. 2017) than our camera grid spacing (1 km^2^). Therefore, individuals could have reasonably been counted at multiple camera sites, creating a proxy layered individual occupancy map for wild pig activity in the study area. We assumed a stable local wild pig population across the study area during the 3‐month study period because there were no hunting or removal efforts of wild pigs during the study period. Furthermore, this timeline was outside peak parturition periods for wild pigs (~20% of annual parturition during 1 October–1 January) and shorter than the length of gestation (~115 days) in wild pigs, so we assumed no significant influence of reproduction on the population during the 3‐month study period (Henry 1968; Ditchkoff et al. 2012; Chinn et al. 2022).

**FIGURE 2:**
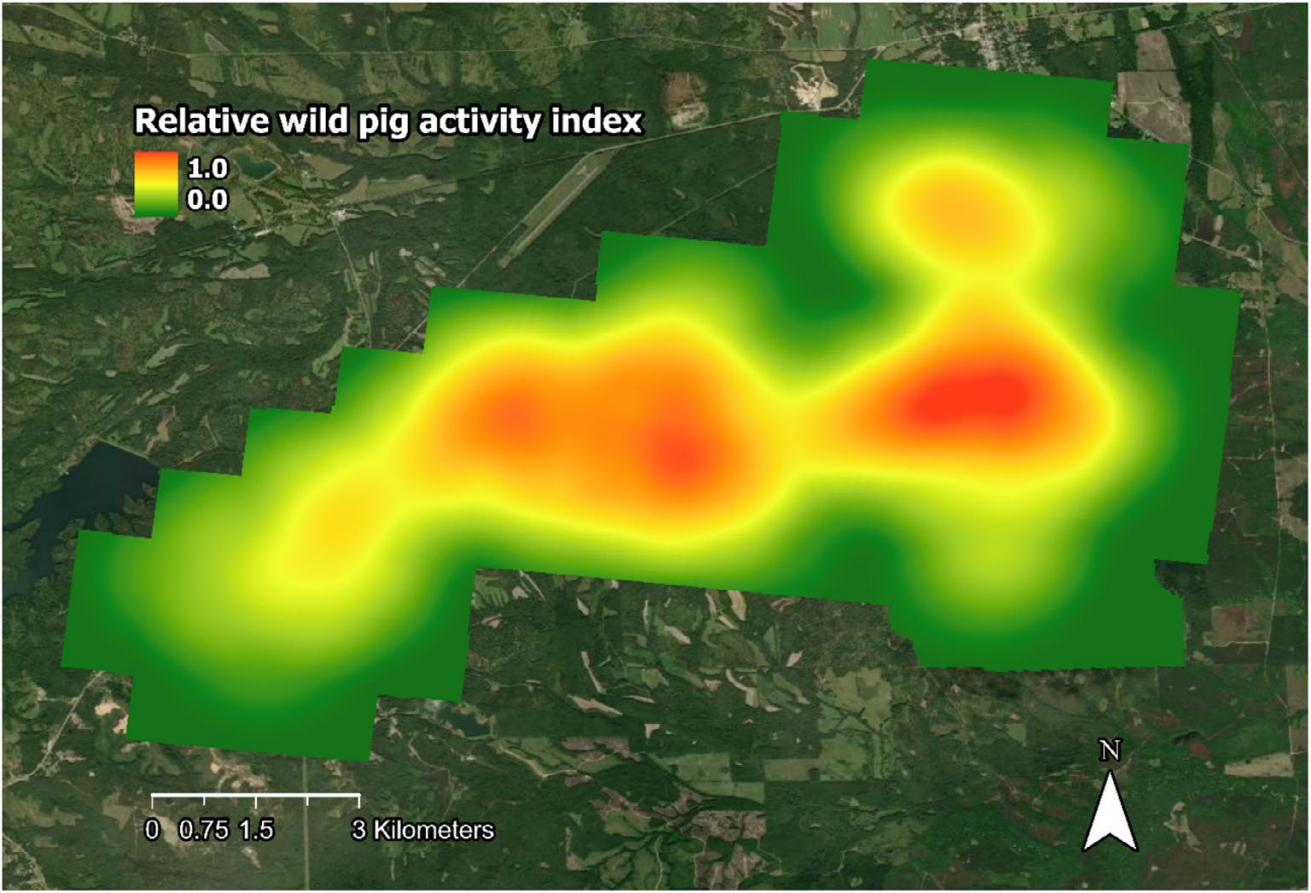
Relative wild pig activity index across buffered study area (0.0–1.0) in east‐central Alabama in 30 m resolution for the October 2022 camera survey. Lower activity indices are shaded green and greater activity indices are shaded red.

Counts of wild pigs were standardized across the study areas as a proxy for relative activity indices per 1 km^2^. Relative activity index values were ranked as percentiles (0.0–1.0/km^2^), ranging from lowest (0.0) to greatest (1.0) activity to account for uncertainty in estimations and allow for more suitable comparisons to other study systems. These percentiles were assigned to respective grid cells in ArcGIS Pro in 1‐km^2^ raster cells via the kernel density estimate tool. We elected to add a 1‐km buffer around the outside of the survey bounds because the average home range of wild pigs that were monitored in the study area was greater than the spacing of our cameras ($\bar{x}$ = 3.45 km^2^, Gomez‐Maldonado n.p.). Buffer values were assigned as the average of adjacent survey cell values. The buffer was omitted for cells bordering the fenced property in the southeastern corner of the study area that were free from wild pigs, as the nature of these cells having no wild pigs should not influence the estimates of wild pigs outside the fence (Figure 2; Machtans et al. 1996; Pollentier et al. 2017; Crawford et al. 2021). Grid cell values were re‐interpolated to 30‐m resolution to match the National Land Cover Data 2021 (NLCD 2021; Figure 1; Dewitz 2021; Figure 2).

### 3.4 Statistical Analysis

We extracted land cover data from NLCD 2021 at 30‐m resolution for land cover types that have known biological importance for wild turkeys: pine forest, hardwood forest, mixed forest, open cover, and riparian (Holbrook et al. 1987; Chance et al. 2020). We delineated pine forest cover type as evergreen (pine) trees occupying > 20% of total vegetation cover with trees > 5‐m tall that retain leaves year‐round, making up > 75% of total tree cover. We delineated hardwood forest cover type as deciduous (hardwood) trees occupying > 20% of total vegetation cover, with trees > 5‐m tall making up > 75% of total tree cover. We delineated mixed forest cover type as trees > 5‐m tall, making up > 20% of total tree cover, with neither evergreen nor deciduous trees making up > 75% of total tree cover. We coded forest cover types of pine, hardwood, and mixed forest as present (1) or absent (0). We combined open cover types of cropland, grassland, shrub, and road cover types into one combined category of open cover, coded as present (1) or absent (0). We created a 100‐m buffer around water and wetland areas for the riparian cover type and classified it as present (1) or absent (0). We removed location fixes outside the study area or with dilution of precision (DOP) > 7 (Bakner et al. 2023). We grouped movement data into the following categories: ALL, FEMALES, and MALES. The ALL grouping consisted of all 12 individual wild turkeys, the FEMALES grouping consisted of the seven females, and the MALES grouping consisted of the five males.

We used a dynamic Brownian bridge movement model in the package move in Program R to calculate movement metrics (Kranstauber et al. 2012; R Core Team 2024). We calculated the Euclidean distance between consecutive points for step length. We extracted relative wild pig activity index values for respective step‐to locations. We used a linear model with a Gaussian distribution to analyze the relationship between step length and wild pig activity for each grouping of wild turkeys (i.e., ALL, MALES, and FEMALES) via the stats package in Program R. We excluded land cover interactions for step length analyses because each step could cross multiple cover types.

We applied a resource selection framework to analyze daytime and roost site selection in relation to wild pig activity. We created a minimum convex polygon (MCP) around each category of points (i.e., all roost site locations for the FEMALES grouping; Johnson 1980). We created 100,000 random locations with ≥ 10 m spacing within the MCP of respective groupings via the create random points function in ArcGIS Pro. For each analysis of daytime use and roost site selection, we included land cover covariates of hardwood forest, mixed forest, pine forest, and riparian cover, each with known importance in wild turkey ecology during autumn and winter (Kilpatrick et al. 1988; Vander Haegen et al. 1989; Burhans et al. 2000; Adey et al. 2023; Gonnerman et al. 2023). The daytime use analysis also included open cover, which was comprised of crops, grasslands, shrubs, and roads land cover types. We used generalized linear models with binomial distributions for daytime use (e.g., ALL, FEMALES, and MALES groupings) and roost site selection (e.g., ALL grouping only) for interactions between land cover variables and estimated wild pig activity to predict probability of use by wild turkeys in respective land cover types via the stats package in Program R. We removed mixed forest from the final model for daytime use due to high collinearity (|*r*| > 0.7) with pine and hardwood forest, as the model with pine or hardwood forest had a better model fit than the model with mixed forest via lower Akaike Information Criterion score. Additionally, we analyzed roost sites for the ALL grouping only due to low sample size of roost locations (i.e., 388 used roost sites). To identify individuals belonging to distinct flocks during the study period, we conducted a proximity analysis via the package geosphere in Program R for all combinations of paired individuals (Hijmans et al. 2017). We set the criterion of shared flock as > 70% of GPS locations within 100 m of another individual at the same time throughout the study period.

## 4 Results

A total of 12 wild turkeys (i.e., five males and seven females belonging to eight distinct flocks) were monitored during the 1 October 2022 to 1 January 2023 study period. Estimates of individual wild pig counts per camera ranged from 0 to 13 pigs/km^2^, which were standardized to relative activity indices (0.0–1.0/km^2^). The average step length was 174.6 m for the ALL grouping, 133.5 m for FEMALES, and 226.4 m for MALES. Wild pig activity was negatively associated with step length for the ALL and MALES groupings (Table 1). For every 10% increase in wild pig activity index, we observed an 11.1 m decrease in step length for the ALL grouping (*p* < 0.001) and a 7.3 m decrease in step length for MALES (*p* = 0.013).

**TABLE 1:** Change in step length (*β* in meters) for every 10% increase in relative activity index of wild pigs ( *Sus scrofa* ) by grouping of wild turkeys ( *Meleagris gallopavo* ) during autumn and winter (study period: 1 October 2022–1 January 2023) in east‐central Alabama.

Grouping	*β*	*p*	95% Confidence interval
ALL	−11.09	< 0.001	−14.37 to −7.82
FEMALES	−1.99	0.243	−5.33 to 1.35
MALES	−7.34	0.013	−13.15 to −1.52

*Note:* The ALL grouping consisted of 12 wild turkeys, FEMALES had 7 individuals, and MALES had 5 individuals.

**FIGURE 3:**
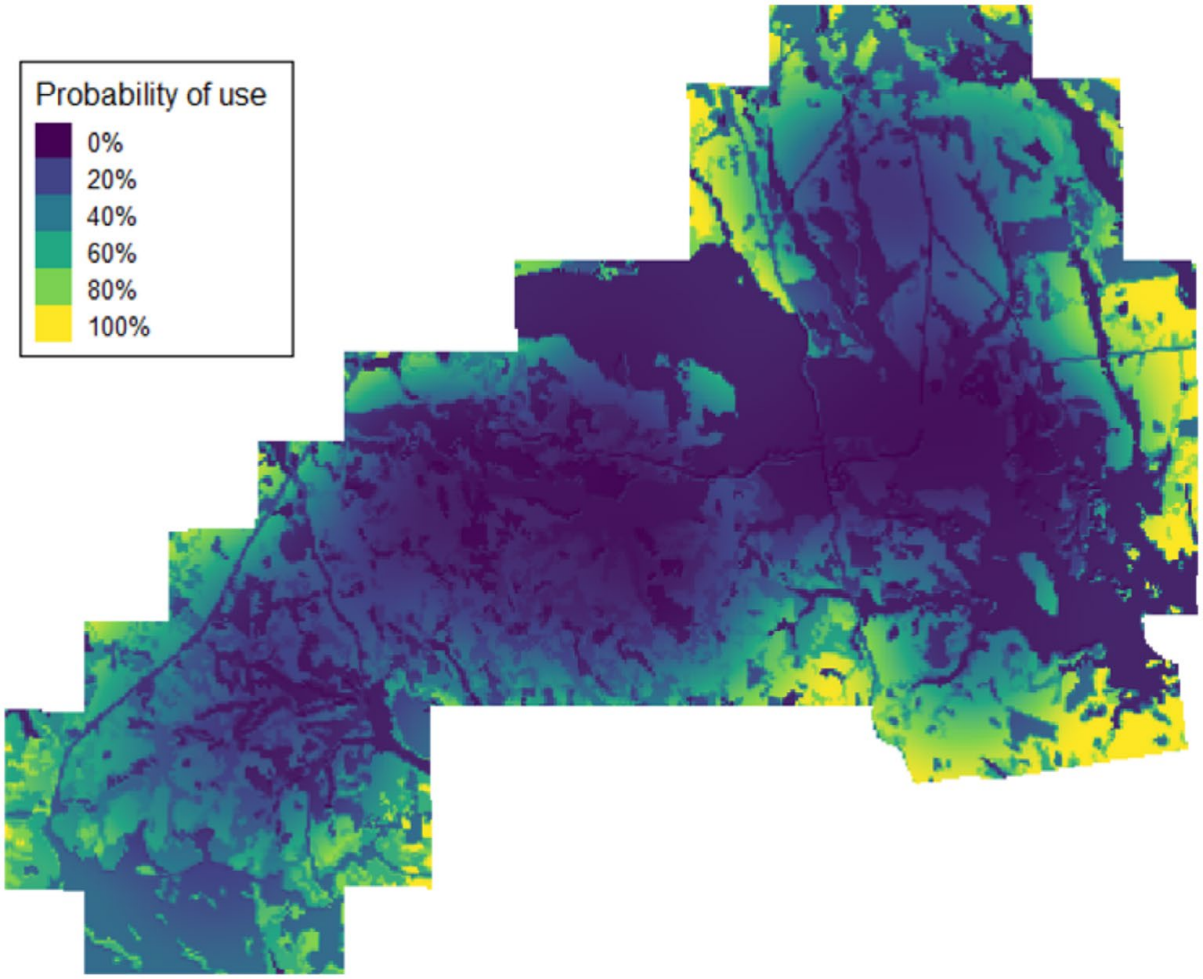
Impact of relative wild pig ( *Sus scrofa* ) activity index and land cover type on the probability of daytime selection for the 12 wild turkeys ( *Meleagris gallopavo* ) in the ALL grouping during autumn and winter (study period: 1 October 2022 to 1 January 2023) in east‐central Alabama. Lower probability of daytime selection was shaded in darker blue and greater probability of daytime selection was shaded in lighter yellow shades.

We recorded 7412 daytime use locations for the ALL grouping of wild turkeys (i.e., 4168 for the FEMALES and 3244 for MALES). Predicted probability of daytime selection by wild turkeys was negatively related to wild pig activity for the ALL (*β* = −2.85, *p* < 0.001), FEMALES (*β* = −2.48, *p* < 0.001), and MALES groupings (*β* = −1.53, *p* < 0.001; Table 2; Figure 3). Additionally, we observed positive relationships between the predicted probability of daytime selection and wild pig activity across multiple land cover types. Specifically, wild pig activity was negatively related to the predicted probability of daytime selection of pine forest cover for ALL (*β* = −0.45, *p* = 0.022), hardwood forest cover for FEMALES (*β* = −3.33, *p* < 0.001), riparian cover for FEMALES (*β* = −9.29, *p* < 0.001), and open cover for ALL (*β* = −0.85, *p* = 0.006). While not statistically significant at the *α* = 0.05 threshold, but potentially of ecological significance, wild pig activity was negatively related to the predicted probability of daytime selection of open cover for FEMALES (*β* = −0.55, *p* = 0.052). We also observed a positive relationship between the predicted probability of daytime selection and wild pig activity across several land cover types. Specifically, wild pig activity was positively related to the predicted probability of daytime selection of pine forest cover for MALES (*β* = 0.79, *p* = 0.005), hardwood forest cover for MALES (*β* = 1.89, *p* < 0.001), and riparian cover for ALL (*β* = 1.87, *p* < 0.001). When examining the wild pig activity index by quantile, we also found a stepwise reduction in the predicted probability of daytime selection by grouping as wild pig activity increased (Table 3). From the least (0.0) to the greatest (1.0) relative wild pig activity index, we observed a decrease in the predicted probability of daytime selection of 16.49% for the ALL grouping, 11.36% for FEMALES, and 5.15% for MALES.

**TABLE 2:** Daytime selection by grouping of wild turkeys ( *Meleagris gallopavo* ) in relation to relative activity index of wild pigs ( *Sus scrofa* ) and landcover interactions during autumn and winter (study period: 1 October 2022–1 January 2023) in east‐central Alabama.

Grouping	Land cover interaction	*β*	Standard error	*p*
ALL	Pig	−2.85	0.175	< 0.001
	Pig*Pine	−0.45	0.195	0.022
	Pig*Hardwood	−0.19	0.243	0.440
	Pig*Riparian	1.87	0.265	< 0.001
	Pig*Open	−0.85	0.307	0.006
FEMALES	Pig	−2.48	0.205	< 0.001
	Pig*Pine	0.38	0.242	0.121
	Pig*Hardwood	−3.33	0.840	< 0.001
	Pig*Riparian	−9.29	0.421	< 0.001
	Pig*Open	−0.55	0.282	0.052
MALES	Pig	−1.53	0.258	< 0.001
	Pig*Pine	0.79	0.786	0.005
	Pig*Hardwood	1.89	0.557	< 0.001
	Pig*Riparian	−0.22	0.339	0.524
	Pig*Open	0.14	0.365	0.696

*Note:* The ALL grouping consisted of 12 wild turkeys; FEMALES had seven individuals, and MALES had five individuals. The direction and significance (*p*) of the *β*‐estimate indicate the log‐odds ratio of effect of the interaction term.

**TABLE 3:** Predicted probability of daytime selection (%) for groupings of wild turkey ( *Meleagris gallopavo* ) by relative activity index quantile (0.0–1.0) of wild pigs ( *Sus scrofa* ) during autumn and winter (study period: 1 October 2022–1 January 2023) in east‐central Alabama.

Grouping	Wild pig activity quantile	Predicted probability of use (%)
ALL	0.00	17.72
	0.25	9.55
	0.50	4.92
	0.75	2.48
	1.00	1.23
FEMALES	0.00	12.55
	0.25	7.17
	0.50	3.99
	0.75	2.19
	1.00	1.19
MALES	0.00	6.67
	0.25	4.64
	0.50	3.21
	0.75	2.21
	1.00	1.52

*Note:* The ALL grouping consisted of 12 wild turkeys, FEMALES had seven individuals, and MALES had five individuals.

**FIGURE 4:**
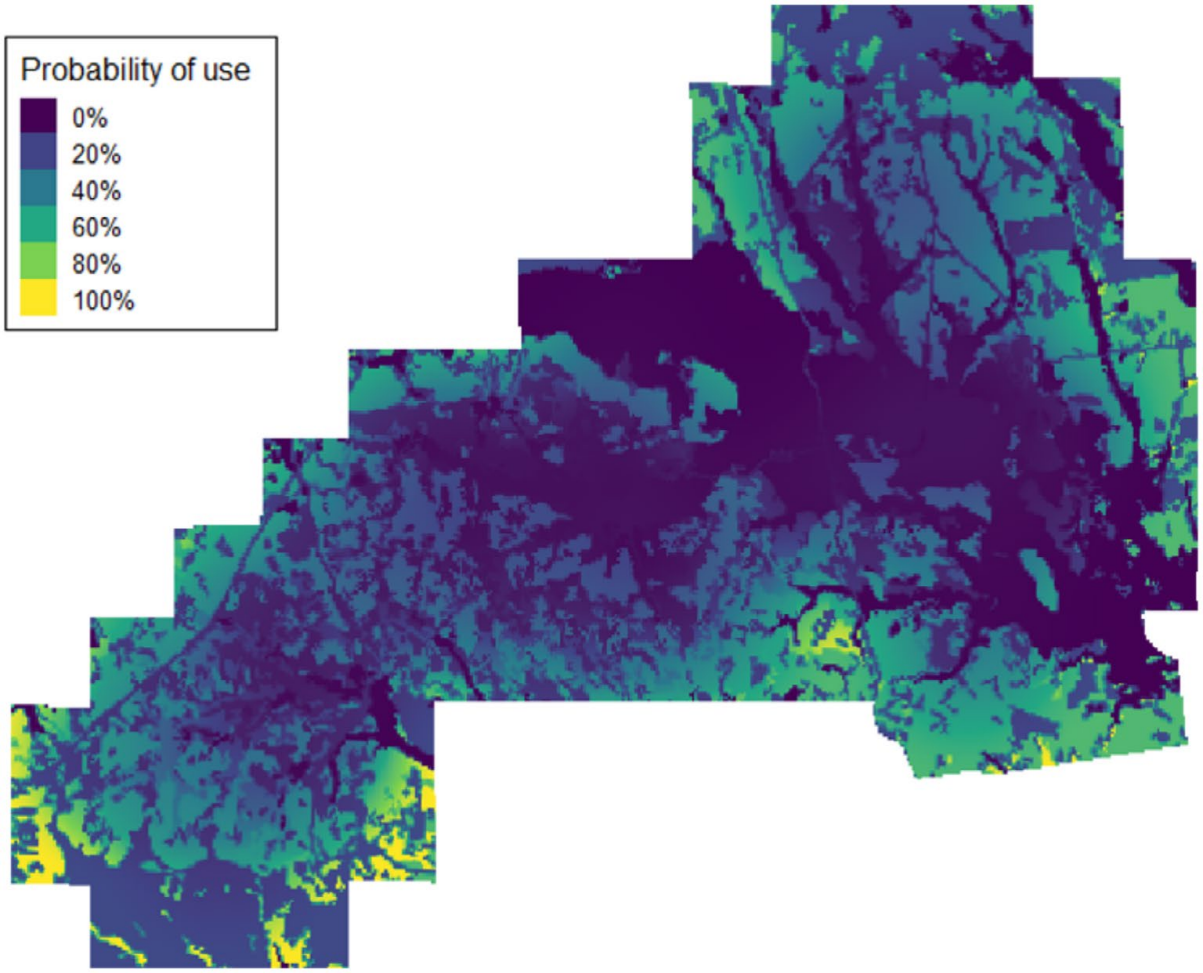
Impact of relative wild pig ( *Sus scrofa* ) activity index and land cover type on the probability of roost site selection for the 12 wild turkeys ( *Meleagris gallopavo* ) in the ALL grouping during autumn and winter (study period: 1 October 2022 to 1 January 2023) in east‐central Alabama. Lower probability of roost site selection was shaded in darker blue and greater probability of roost site selection was shaded in lighter yellow shades.

We recorded 338 roost site locations for the ALL grouping of wild turkeys (i.e., 194 for the FEMALES and 144 for MALES). We observed a negative predicted probability of roost site selection in relation to wild pig activity (*β* = −1.81, *p* = 0.013; Table 4; Figure 4). While not statistically significant at the *α* = 0.05 threshold, but potentially of ecological significance, wild pig activity was also negatively related to the predicted probability of roost site selection of hardwood forest cover (*β* = −1.78, *p* = 0.071). Conversely, we found a positive relationship between wild pig activity and roost site selection of riparian cover (*β* = 2.76, *p* = 0.020). When examining wild pig activity by quantile, we did not find a clear stepwise reduction in the predicted probability of roost site selection by grouping as wild pig activity increased (Table 5). From the least (0.0) to the greatest (1.0) relative wild pig activity index, we observed a decrease in the predicted probability of roost site selection of only 0.27%.

**TABLE 4:** Roost site selection for 12 wild turkeys ( *Meleagris gallopavo* ) in relation to relative activity index of wild pigs ( *Sus scrofa* ) and landcover interactions during autumn and winter (study period: 1 October 2022–1 January 2023) in east‐central Alabama.

Grouping	Land cover interaction	*β*	Standard error	*p*
ALL	Pig	−1.81	0.727	0.013
	Pig*Pine	0.46	0.789	0.558
	Pig*Hardwood	−1.78	0.986	0.071
	Pig*Riparian	2.76	1.187	0.020

*Note:* The direction and significance (*p*) of the *β*‐estimate indicate the log‐odds ratio of effect of the interaction term.

**TABLE 5:** Predicted probability of roost site selection (%) for 12 wild turkeys ( *Meleagris gallopavo* ) by relative activity index quantile (0.0–1.0) of wild pigs ( *Sus scrofa* ) during autumn and winter (study period: 1 October 2022 to 1 January 2023) in east‐central Alabama.

Grouping	Wild pig activity quantile	Predicted probability of use (%)
ALL	0.00	0.32
	0.25	0.20
	0.50	0.13
	0.75	0.08
	1.00	0.05

## 5 Discussion

Our results suggest that wild pig activity was related to several metrics of movement and space use of wild turkeys during the autumn and winter seasons. Specifically, we observed increases in step length and lower predicted probabilities of daytime use and roost site selection in areas with greater wild pig activity. Evidence from camera‐based studies has supported a negative temporal relationship between wild pig activity and detection of wild turkeys (Walters and Osborne 2022; Smith 2023); however, the spatial relationship has not been well‐examined. Lewis (2021) conducted baited camera surveys for wild turkeys and reported that the probability of use of a site increased by 11% and detection increased by 9% when wild pig sounders were absent. McDonough et al. (2024) found that 100% removal of the original baseline estimate of wild pigs led to an average of 2.0 times the detection and 1.5 times the population estimate of wild turkeys compared to prior to removal. Similar results were found for the Lord Howe Island woodhen ( *Tricholimnas sylvestris* ), in which areas that underwent wild pig removal were observed increased use by woodhens (Miller and Mullette 1985). Wild pigs posed some predatory risk to woodhens, and though adult wild turkeys are not considered to be prey for wild pigs, we believe that wild turkeys could potentially avoid wild pigs due to perceived risk.

A negative relationship between daytime selection and wild pig activity was also demonstrated in hardwood forest and riparian cover for the FEMALES groupings, which is potentially explainable by competition for hard mast. Hard mast (e.g., acorns) is spatiotemporally constrained to deciduous‐dominated areas like hardwood stands and streamside management zones (i.e., riparian areas) during autumn and winter (Godfrey 1988; McWilliams 1992). Acorns are an integral forage for wild turkeys during the autumn and winter seasons due to high fat content and metabolizable energy, comprising 20%–33% of the total diet during the autumn and winter (Ellis and Lewis 1967; Zwank et al. 1988; McShea and Healy 2003). As such, limited access to hard mast in the months leading up to the reproductive season may have negative effects on condition, production, and survival in spring (Porter et al. 1983; Vangilder and Kurzejeski 1995; McShea and Healy 2003; Lehman et al. 2010). Acorns are also important food sources for wild pigs (Ditchkoff and Mayer 2009; Schlichting et al. 2015), sometimes comprising > 75% of total dietary dry mass during autumn and winter (Wood and Roark 1980). Fay et al. (2023) conducted a camera survey baited with acorns from October 2018 to February 2019 and found that wild pigs consumed the greatest percentage of acorns of any species (23%). Competition for a spatiotemporally limited forage like hard mast can lead to reduced rates of consumption and increased use of other foraging areas (MacArthur 1958; Walter 1991). We believe the consumption of hard mast by wild pigs could reduce the availability of hard mast, which led to reduced use of hardwood areas by female wild turkeys in our study.

In addition to observing a negative association of some wild turkeys with wild pigs in hardwood and riparian areas, we found a positive association with wild pigs in several other land cover types. Multiple studies reported that wild turkeys shift resource use away from hardwood forests when hard mast is limited (Vander Haegen et al. 1989; Roberts et al. 1995; McShea and Healy 2003). Wild pigs may reduce hard mast availability (Fay et al. 2023), particularly in hardwood areas, which may lead to increased selection of other cover types by wild turkeys. The effects of wild pigs on wild turkeys may also be different in hardwood areas than in other cover types. Walters and Osborne (2022) examined occurrence patterns of wild turkey in relation to wild pigs and suggested that the presence of wild pigs in an area may exclude or alter space use of wild turkeys, leading to increased use of land cover types by wild turkeys that they would otherwise not typically use in the absence of wild pigs.

We also observed a negative relationship between wild pig activity and roost site selection for wild turkeys. Roost sites are typically mature hardwood and pine trees with branches 3‐ to 10‐m high (Austin and Degraff 1975; Kilpatrick et al. 1988). Avoidance of predation risk and environmental exposure has been suggested as a primary attribute in the selection of suitable roost sites among wild turkeys (Adey et al. 2023; Gonnerman et al. 2023). During the evening, when roost sites are selected (Adey et al. 2023), wild pig activity is generally high (Clontz et al. 2021; Garabedian et al. 2023). Wolfson et al. (2023) reported that ≥ 70% of activity by wild pigs occurred during crepuscular and nighttime periods during autumn and winter. Wild turkeys also have poor vision during crepuscular and nighttime periods and thus could be at greatest risk of disturbance or predation during this time (Miller 2018). Although wild pigs are not considered to be predators of adult wild turkeys, wild turkeys may still avoid areas with high wild pig activity to minimize perceived risk (Stankowich and Blemstein 2005). Wild turkeys also avoided areas with high wild pig activity during the daytime, so it is possible that this led to avoidance when selecting a roost site.

We observed reduced step lengths by wild turkeys in areas with greater wild pig activity for the ALL and MALES groupings. We believe that the selfish herd hypothesis as proposed by Hamilton (1971) and the abatement effect hypothesis as proposed by Turner and Pitcher (1986) could help explain these seasonal differences. The selfish herd hypothesis suggests that gregarious behaviors help to improve foraging efficiency and reduce the risk of predation (Hamilton 1971; Morton et al. 1994; Quinn and Cresswell 2006; Hammer et al. 2023). Similarly, the abatement effect hypothesis suggests that forming cohesive groups can decrease predation and disturbance by increasing dilution and vigilance (Turner and Pitcher 1986; Warburton and Lazarus 1991; Wrona and Dixon 1991; Viscido et al. 2001). Wild turkeys are a sentinel gregarious species that flock during the autumn and winter but separate at the initiation of the spring breeding season (Watts and Stokes 1971; Vangilder and Kurzejeski 1995). Measures of step length during the autumn and winter are therefore more than a representation of the movement of an individual but also the movement of the flock. The response of an individual (such as during the spring breeding period) to risk may be to escape and increase the rate or linearity of movement (Lima and Dill 1990; James and Stuart‐Smith 2000; Prokopenko et al. 2017; Thompson et al. 2024). Alternatively, the response of the flock could be to reduce speed and decrease the linearity of movement to increase vigilance (Watts and Stokes 1971; Hatle and Grimké Faragher 1998; Persons et al. 2001; Sabal et al. 2020; Wirsing et al. 2021).

Our study provides a novel methodological framework for testing the cooperation of camera‐based surveys and GPS‐movement data to examine a potential spatiotemporal relationship between an invasive omnivorous ungulate and a declining native ground‐nesting bird. We recognize the value of our methodological framework for applications to other study systems such as predator–prey interactions or inference competition. However, we also recognize the evaluative limitations of the short duration of our study period, the more basic sample size of GPS‐tagged wild turkeys, and the use of a single contiguous study area. For example, we used a seven‐day camera survey during the 3‐month study period to snapshot relative activity of wild pigs. Recent research has suggested that wild pig sounders exhibit high levels of site fidelity over time (Ditchkoff and Bodenchuk 2020; Lewis et al. 2022; Evans et al. 2024), supporting the use of our camera survey design. However, we acknowledge that the relative activity of wild pigs measured during the camera survey could have potentially shifted during the study period, as we did not directly measure activity over the entire study period. We suggest future research could more directly evaluate potential spatiotemporal relationships utilizing our methodological framework via a multi‐season and multi‐year study period and the use of multiple disconnected study areas with a greater sample size of GPS‐tagged individuals.

## Author Contributions

**Travis E. Stoakley:** conceptualization (equal), data curation (lead), formal analysis (lead), methodology (equal), writing – original draft (lead), writing – review and editing (equal). **Stephen J. Zenas:** conceptualization (equal), methodology (equal), project administration (equal), writing – review and editing (equal). **Vienna R. Brown:** conceptualization (equal), funding acquisition (equal), writing – review and editing (equal). **Mark D. Smith:** conceptualization (equal), methodology (equal), writing – review and editing (equal). **William D. Gulsby:** conceptualization (equal), methodology (equal), writing – review and editing (equal). **Bret A. Collier:** conceptualization (equal), methodology (equal), project administration (equal), writing – review and editing (equal). **Stephen S. Ditchkoff:** conceptualization (equal), funding acquisition (equal), investigation (equal), methodology (equal), project administration (lead), writing – review and editing (equal).

## Ethics Statement

All animal handling practices were approved by Auburn University IACUC (PRN: 2021‐3994).

## Conflicts of Interest

The authors declare no conflicts of interest.

## Data Availability

The data that support the findings of this study are openly available in the Harvard Dataverse repository (doi.org/10.7910/DVN/SXWCHY).
